# Application of multispectral imaging in quantitative immunohistochemistry study of breast cancer: a comparative study

**DOI:** 10.1007/s13277-015-4327-9

**Published:** 2015-11-05

**Authors:** Wen-Lou Liu, Lin-Wei Wang, Jia-Mei Chen, Jing-Ping Yuan, Qing-Ming Xiang, Gui-Fang Yang, Ai-Ping Qu, Juan Liu, Yan Li

**Affiliations:** 1 Department of Oncology, Zhongnan Hospital of Wuhan University, Hubei Key Laboratory of Tumor Biological Behaviors & Hubei Cancer Clinical Study Center, Wuhan, 430071 China; 2 Department of Pathology, Zhongnan Hospital of Wuhan University, Wuhan, 430071 China; 3 Key State Laboratory of Software Engineering, School of Computer, Wuhan University, Wuhan, 430072 China; 4 Department of Peritoneal Cancer Surgery, Beijing Shijitan Hospital of Capital Medical University, Beijing, 100038 China; 5 Department of Oncology, Zhongnan Hospital of Wuhan University; Department of Peritoneal Cancer Surgery, Beijing Shijitan Hospital of Capital Medical University, No 10, Tieyi Road, Yangfangdian, Haidian District, Beijing, 100038 China

**Keywords:** Invasive breast cancer, Multispectral imaging, Immunohistochemistry, Quantitative image analysis, Optical density

## Abstract

Multispectral imaging (MSI) based on imaging and spectroscopy, as relatively novel to the field of histopathology, has been used in biomedical multidisciplinary researches. We analyzed and compared the utility of multispectral (MS) versus conventional red–green–blue (RGB) images for immunohistochemistry (IHC) staining to explore the advantages of MSI in clinical-pathological diagnosis. The MS images acquired of IHC-stained membranous marker human epidermal growth factor receptor 2 (HER2), cytoplasmic marker cytokeratin5/6 (CK5/6), and nuclear marker estrogen receptor (ER) have higher resolution, stronger contrast, and more accurate segmentation than the RGB images. The total signal optical density (OD) values for each biomarker were higher in MS images than in RGB images (all *P* < 0.05). Moreover, receiver operator characteristic (ROC) analysis revealed that a greater area under the curve (AUC), higher sensitivity, and specificity in evaluation of HER2 gene were achieved by MS images (AUC = 0.91, 89.1 %, 83.2 %) than RGB images (AUC = 0.87, 84.5, and 81.8 %). There was no significant difference between quantitative results of RGB images and clinico-pathological characteristics (*P* > 0.05). However, by quantifying MS images, the total signal OD values of HER2 positive expression were correlated with lymph node status and histological grades (*P* = 0.02 and 0.04). Additionally, the consistency test results indicated the inter-observer agreement was more robust in MS images for HER2 (inter-class correlation coefficient (ICC) = 0.95, *r*_s_ = 0.94), CK5/6 (ICC = 0.90, *r*_s_ = 0.88), and ER (ICC = 0.94, *r*_s_ = 0.94) (all *P* < 0.001) than that in RGB images for HER2 (ICC = 0.91, *r*_s_ = 0.89), CK5/6 (ICC = 0.85, *r*_s_ = 0.84), and ER (ICC = 0.90, *r*_s_ = 0.89) (all *P* < 0.001). Our results suggest that the application of MS images in quantitative IHC analysis could obtain higher accuracy, reliability, and more information of protein expression in relation to clinico-pathological characteristics versus conventional RGB images. It may become an optimal IHC digital imaging system used in quantitative pathology.

## Introduction

Immunohistochemistry (IHC) has become a potent approach for detecting antigens in tissues. However, in current clinical pathology practice, interpreting IHC images is largely based on the personal experience of the expert pathologist, which is the major cause of different conclusions on the same IHC image among the pathologists, and such inter- and intra-observer variations could pose significant difficulties to clinical treatment-decision making [1, 2]. Therefore, in the era of digital pathology, how to improve the accuracy and repeatability of quantitative analysis of IHC images and minimize the personal errors remains a formidable task [2]. Previous study showed that image quality and color variations directly affect the result of image analysis made by pathologist or computer-aided image analysis [3].

The conventional red–green–blue (RGB) images by common imaging technology are difficult to accurately distinguish labeled targets in the specific target region, due to inherent technical limitations including suboptimal image contrast and sharpness, strong light effect, overlapping chromogens, and varied staining intensities within or between chromogens, which directly affect the accuracy and repeatability in IHC image quantification and analysis [4, 5]. A solution to this problem has to be found so as to improve the current IHC image reading and reporting system. However, an effective method to overcome this limitation is to use a multispectral imaging (MSI) system to acquire images and perform linear unmixing to separate and quantify each marker without interference from the others, irrespective of image overlap spatially [4–6].

Recent developments in MSI systems such as the Nuance™ MSI system have greatly facilitated the imaging, visualization, and quantitative analysis of single-color or multicolor tissue samples, both in bright-field images and fluorescent images [7, 8]. The advantages of MSI over traditional RGB imaging are to transform black–white or color RGB images with low contrast into spectral images with high color dimension, and obtain high-resolution images with precise spectral and spatial information [9, 10]. Such spectral images could facilitate the extraction of specific chromogen information from the bright-field image according to spectroscopy principle, and such technical advantage could optimally remove background auto-fluorescence from the sample and significantly increase the signal-to-noise ratio (SNR) of the image to enhance the image quality for subsequent analysis [10, 11].

In routine clinical histopathology, however, it remains unknown whether the application of MS images in quantitative IHC analysis could obtain higher sensitivity and specificity, although some promising results of MSI in automated quantitative scoring of IHC have been reported [7, 8, 12, 13]. In this study, we used invasive breast cancer (BC) IHC images as the analysis object, and HER2, CK5/6, and ER were selected for IHC staining to represent interesting biomarkers at the cellular membrane, cytoplasm, and nucleus, respectively. The objective of our study is to investigate if the automated quantification of MS images of the biomarkers could increase the precision and reliability of image analysis and provide more information compared with conventional RGB images, and to develop a standard digital imaging system for IHC diagnosis in clinical pathology.

## Materials and methods

### Patients and tumor samples preparation

**Fig. 1:**
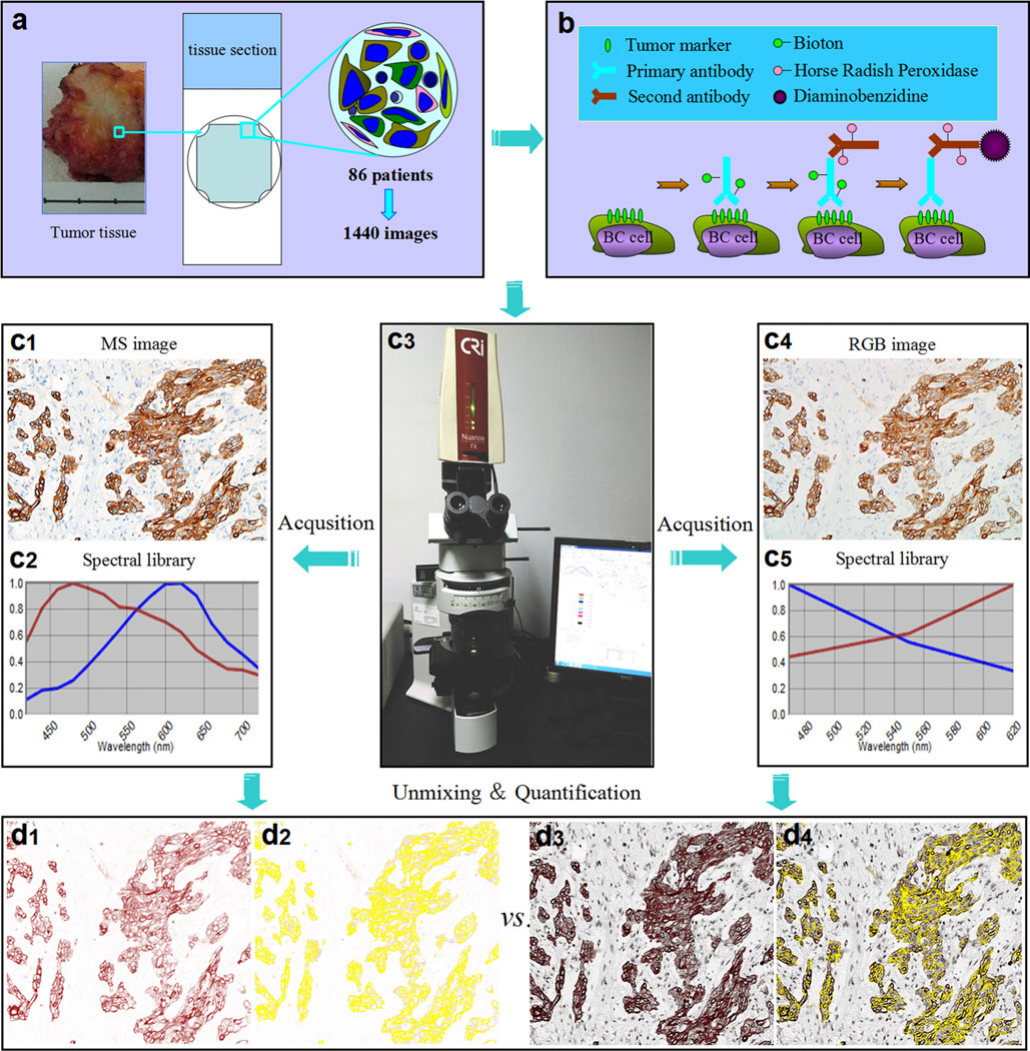
The design and major technical procedures of this study. **a** Tumor tissue section slides were prepared from 86 selected patients with invasive BC. **b** Section slides were stained with three biomarkers in routine IHC, staining HER2 in cell membrane, CK5/6 in cytoplasm, and ER in cell nucleus. **c1–c5** The MS (**c1**) and RGB images (**c4**) were captured and its spectral curves (**c2**, **c5**) were obtained by CRi Nuance software. **d1–d4** The positive regions in the MS images (**d1**, **d2**) and RGB images (**d3**, **d4**) were unmixed and quantified by CRi Nuance software, respectively, and the quantitative results of MS and RGB images were compared. BC: breast cancer; HER2: human epidermal growth factor receptor-2; CK5/6: cytokeratin5/6; ER: estrogen receptor

The study protocol was approved by the Institutional Ethics Committee of Zhongnan Hospital of Wuhan University and informed consent was obtained from all patients. The study was undertaken according to the ethical standards of the World Medical Association Declaration of Helsinki. Formalin-fixed, paraffin-embedded (FFPE) tissue breast cancer (BC) specimens were obtained from our prospectively established cancer database, which has been the source of information for several studies [14–16]. From this database, 86 patients with invasive BC between 2009 and 2010 were selected for this study. Major clinico-pathological parameters including age, menopausal status, the tumor size, lymph node status, histological grade, ER, and HER2 gene status are summarized in Table 1. TNM staging and histological grade were classified according to the seventh edition of American Joint Committee on Cancer (AJCC) TNM system [17] and the fourth edition of WHO histological grade [18]. Serial tissue sections (4-μm thickness) of invasive BC were prepared for IHC staining before proceeding to the following imaging studies (Fig. 1a).

**Table 1:** Clinico-pathological characteristics of 86 breast cancer patients

Characteristics	Value (%)
Age (years)	
<50	32 (37.2)
>50	54 (62.8)
Menopausal status	
Premenopausal	29 (33.7)
Postmenopausal	57 (66.3)
Tumor size (cm)	
T1 (T ≤ 2)	9 (10.5)
T2 (2 < T ≤ 5)	61 (70.9)
T3 (T > 5)	16 (18.6)
Lymph node status	
Positive	36 (41.9)
Negative	50 (58.1)
Histological grades	
І	17 (19.8)
П	46 (53.5)
Ш	23 (26.7)
ER status^a^	
Positive	40 (46.5)
Negative	46 (53.5)
CK5/6 expression	
Positive	35 (40.7)
Negative	51 (59.3)
HER2 expression^a^	
Positive (IHC 3+/2+)	45 (19/26) (52.3)
Negative (IHC 0-1+)	41 (47.7)
HER2 gene status^b^	
Amplification	28 (32.6)
Non-amplification	58 (67.4)
Chemotherapy	
No	37(43.0)
Yes	49(57.0)
Recurrence	
No	51(59.3)
Yes	35(40.7)

^a^ER and HER2 was determined by immunohistochemical staining according to guideline [21]

^b^HER2 gene was determined by fluorescent in situ hybridization (FISH) according to guideline [22]

*HER2* human epidermal growth factor receptor-2, *CK5/6* cytokeratin5/6, *ER* estrogen receptor

### Immunohistochemistry staining and evaluation

Immunostaining of HER2, CK5/6, and ER was performed using streptavidin-biotin peroxidase complex method (SP). Major IHC procedures were described previously [19]. In short (Fig. 1b), tissue slides were first deparaffinized in xylene, ethanol, and water, and then the slides were pretreated in 0.01 M citrate buffer (pH 6.0) and heated in a microwave oven (95 °C) for 15 min. After cooling at room temperature, endogenous peroxidase activity was quenched by immersing in 3 % H_2_O_2_ for 10 min to eliminate nonspecific binding. Then, blocked with 2 % bovine serum albumin (BSA), the slides were first incubated with primary antibodies of mouse anti-human polyclonal antibody against HER2 (A-0485, Dako, Denmark, 1:50 dilution), mouse anti-human monoclonal antibody against CK5/6 (ab-86974, Abcam, England, 1:150 dilution), and rabbit anti-human monoclonal antibody against ER (sc-7207, Santa Cruz, USA, 1:100 dilution) for 2 h at 37 °C and then incubated with the corresponding secondary antibody (Maixin Biotechnology, China, 1:250 dilution) for 30 min at 37 °C. Primary antibody was replaced with Tris-buffered saline on section as negative control. The reaction products were visualized with diaminobenzidine (DAKO, Denmark). All slides were treated with 0.2 % diaminobenzidine (DAB, DAKO, Denmark) solution for 2 min to develop, counterstained with hematoxylin, and differentiated by hydrochloric acid alcohol.

The immunostaining results of three biomarkers were interpreted independently by two expert pathologists (GF Yang, JP Yuan) in clinical laboratories based on the most updated pathological guidelines [20]. Tissue slides that were lost or damaged during sectioning were not scored, and the number of evaluable cases for each analysis is indicated in Table 1. In this study, HER2 membranous staining was scored from 0 to 3+ according to the established criteria [20], and the data for HER2 staining were categorized into negative (0 and 1+) versus positive (>3+) cases. Cases with HER2 2+ or 3+ score were further analyzed for HER2 gene amplification by fluorescence in situ hybridization (FISH) technique [21], and major procedures of FISH were previously described [22]. For CK5/6, more than 10 % of the tumor cells with immunostaining along the cellular periphery and/or in the cytoplasm were defined as positive [23]. The data for ER staining were dichotomized into negative (<1 %) versus positive (>1 %) cases [20].

### Image acquisition

The digital images were acquired under an Olympus BX52 bright-field microscope equipped with an Olympus DP72 RGB camera (Olympus Optical Co., Ltd., Tokyo, Japan) and CRi Nuance multispectral imaging systems (Cambridge Research & Instrumentation, Inc., Woburn, MA, USA) respectively by the following steps. First, the regions of interests (ROIs) in the IHC images with characteristic invasive BC were selected at ×100; the necrotic area and ductal carcinoma in situ were excluded. Second, each ROI containing both tumor nests and stroma was captured at ×400 following the unified image acquisition parameters (e.g., ×400 lens magnification, constant light source, autofocus, and exposure time) by two imaging systems. To minimize selection bias, six high-power field (HPF) images per slide were randomly selected from the ROI. For each IHC staining section, a total of six spectral cubes containing the complete spectral information at 20-nm wavelength intervals from 420 to 720 nm were randomly obtained from different areas of the section by CRi Nuance multispectral imaging systems. As a result, 1440 bright-field IHC images of three biomarkers were captured including identical 720 RGB images and 720 MS images, and all saved in Tagged Image File Format (TIFF) with consistent resolution of 1360 × 1024 pixels for analysis.

### Quantitative image analysis

After imaging acquisition, the software package within CRi Nuance multispectral imaging system was used for quantification in MS and RGB images of IHC-positive areas of HER2, CK5/6, and ER with standard image unmixing and processing method. The results were analyzed twice by two expert pathologists (YGF, YJP); all users train with the software to recognize tumor versus stroma. CRi Nuance 3.0.0 software was used to build the spectral libraries and to extract spectral information in bright-field MS (Fig. 1c1) and RGB (Fig. 1c4) images with a cube format (RGB images were converted to cube format). Each chromogen has its unique spectral characteristics (curve), which is the basis for building the spectral library. The spectral characteristics (curve) for two chromogens (DAB and hematoxylin) in MS (Fig. 1c2) and RGB (Fig. 1c5) images were obtained by spectral unmixing. The spectral library was built from the control slides, which was used to unmix the positive signals (DAB) on each test slide by recognizing its unique spectral curves for quantification. Specific steps of CRi Nuance software were described [24] and briefed as the following: (1) selection of targets of HER2, CK5/6, ER, and background with different spectra; (2) image unmixing and elimination of background crosstalk: the target images with different spectra were automatically unmixed into DAB (molecular status) (Fig. 1d1, d3) and hematoxylin component images by the software based on spectral library; and (3) quantification of unmixed DAB component images (Fig. 1d2, d4). HER2, CK5/6, and ER expression levels stained on IHC slide were best quantitated as total optical density (OD) values per unit of positive area in one view field. The final quantitative results of each biomarker were the average values of the six cube images.

### Statistical analysis

All numeric values are reported as the mean ± standard deviation, and Student’s *t* test of paired data was used to compare the quantitative results of IHC RGB and MS images of three biomarkers. Receiver operating characteristic (ROC) curve analysis was used to compare the accuracy of analysis outputs of RGB and MS images for HER2 gene using FISH results as gold standard. Inter-observer variation between the quantitative analysis results of RGB and MS images of three biomarkers was determined by the use of inter-class correlation coefficient (ICC) and Spearman’s correlation (*r*_s_). ICC ≥ 0.6 is considered to be acceptable, and >0.7 as good. The one-way analysis of variance or Student’s *t* test was used to analyze the correlations between different image information and the clinico-pathological features. All statistical analyses were performed using the SPSS Statistics 19.0 software program (SPSS Institute, Chicago, IL, USA). Two-sided *P* < 0.05 was considered as statistically significant.

## Results

### IHC staining results and imaging quality

**Fig. 2:**
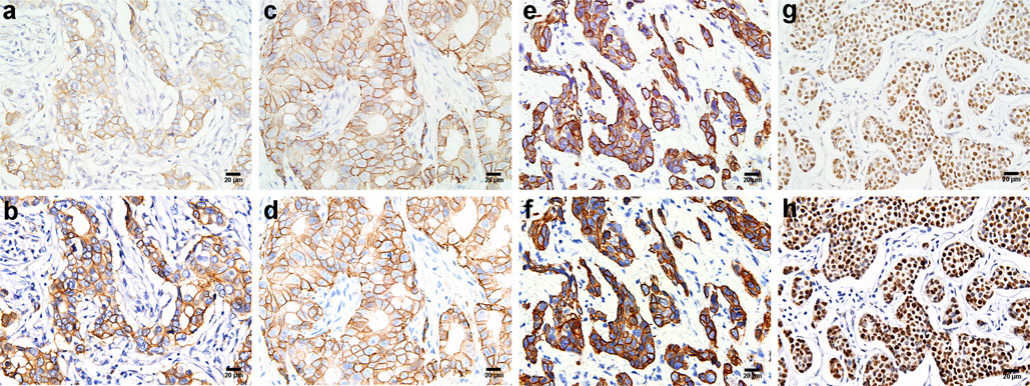
Representative images of immunostaining for three molecular biomarkers. **a–g** The RGB images acquired by RGB imaging system. **b–h** The corresponding MS images acquired by MSI system. **a**, **b** Positive HER2 membrane staining was scored as 2+. **c**, **d** Positive HER2 membrane staining was scored as 3+. **e**, **f** CK5/6 staining mainly in the cytoplasm; this tumor was classified as CK5/6-positive. **g**, **h** ER staining in the nucleus, this tumor was scored as ER-positive. The magnification of all figures is ×400. *Scale bar*: 20 μm for all images

The expression of HER2, CK5/6, and ER in BC specimens was studied by IHC. The positive staining was brown or brownish granules, HER2 was expressed in cell membrane, CK5/6 was mainly in cell cytoplasm, and ER in cell nucleus. The overall staining was clear and there was no background “noise” in the IHC staining. Among the 86 patients in this study, HER2 was expressed in 45 cases (52.3 %), CK5/6 in 35 cases (40.7 %), and ER in 40 cases (46.5 %, Table 1). Identical RGB and MS images of these biomarkers were acquired by conventional RGB imaging and MSI system, with representative IHC images of these markers shown in Fig. 2. Compared with the RGB images, MS images for each marker had higher sharpness, stronger contrast, better white balance processing and color reduction, and more effective removal of tissue spontaneous light effect, thus ensuring better image quality for computer analysis.

#### Unmixing effect of the RGB and MS images

**Fig. 3:**
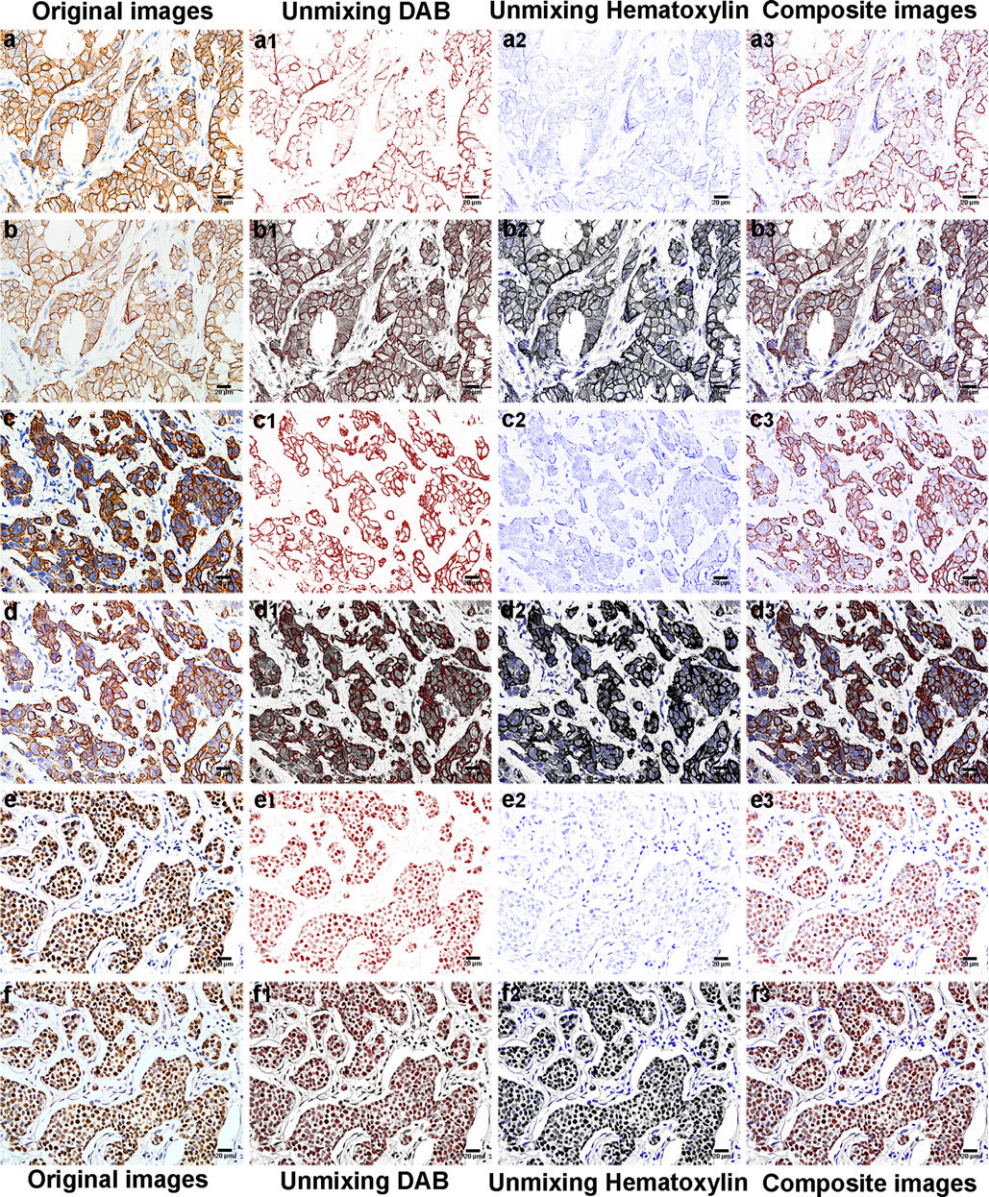
MS and RGB images of BC tissues with IHC staining and images of unmixed signals. **a–f** The original MS and RGB images of membrane HER2 (**a**, **b**), cytoplasm CK5/6 (**c**, **d**), and nucleus ER (**e**, **f**). **a1**–**f3** Unmixed DAB, hematoxylin signal, and composite images of MS images (**a1**–**a3**) and RGB images (**b1**–**b3**) of HER2, MS images (**c1**–**c3**) and RGB images (**d1**–**d3**) of CK5/6, and MS images (**e1**–**e3**) and RGB images (**f1**–**f3**) of ER. The magnification of all figures is ×400. *Scale bar*: 20 μm for all images

The spectral library was used to unmix the positive signals (DAB) and the hematoxylin signal on each test slide by recognizing its unique spectral curves for quantification. After unmixing the MS images, every individual unmixed single-channel and composite images thus obtained for three markers could show clean background, strong object contrast, and clear positive object silhouette, no matter whether the positive choromogens are in the membrane (Fig. 3a1–a3), the cytoplasm (Fig. 3c1–c3), or the nucleus (Fig. 3e1–e3). In contrast, every individual unmixed single-channel and composite images obtained from unmixing RGB images of three markers could not show clear object segmentation and effective removal of background signal noise, no matter whether the positive choromogens are in the membrane (Fig. 3b1–b3), the cytoplasm (Fig. 3d1–d3), or the nucleus (Fig. 3f1–f3).

### Quantitative image analysis

The total signal OD values in RGB and MS images of membrane (HER2), cytoplasm (CK5/6), and nucleus (ER) were measured by CRi Nuance software. All the total signal OD values of HER2, CK5/6, and ER markers are significantly higher in MS images than in RGB images, with statistically significant differences between them (87.510 ± 39.885 vs. 99.833 ± 50.829, *P* < 0.05; 69.917 ± 26.210 vs. 76.205 ± 29.771, *P* < 0.05; 64.879 ± 24.932 vs. 70.989 ± 27.958, *P* < 0.05, respectively, Table 2).

**Table 2:** Quantitative image analysis of HER2, CK5/6, and ER by CRi Nuance software

Marker	Images (*n*)	Total signal OD values (×10^3^)		*T* value	*P* value
		RGB images	MS images		
HER2	540	87.510 ± 39.885	99.833 ± 50.829*	−13.557	0.000
CK5/6	420	69.917 ± 26.210	76.205 ± 29.771*	−12.641	0.000
ER	480	64.879 ± 24.932	70.989 ± 27.958*	−13.482	0.000

Data were presented as means ± standard deviation

*OD* optical density

* *P* < 0.05 compared with RGB images

#### Comparisons in HER2 expression among RGB images, MS images, and FISH

**Fig. 4:**
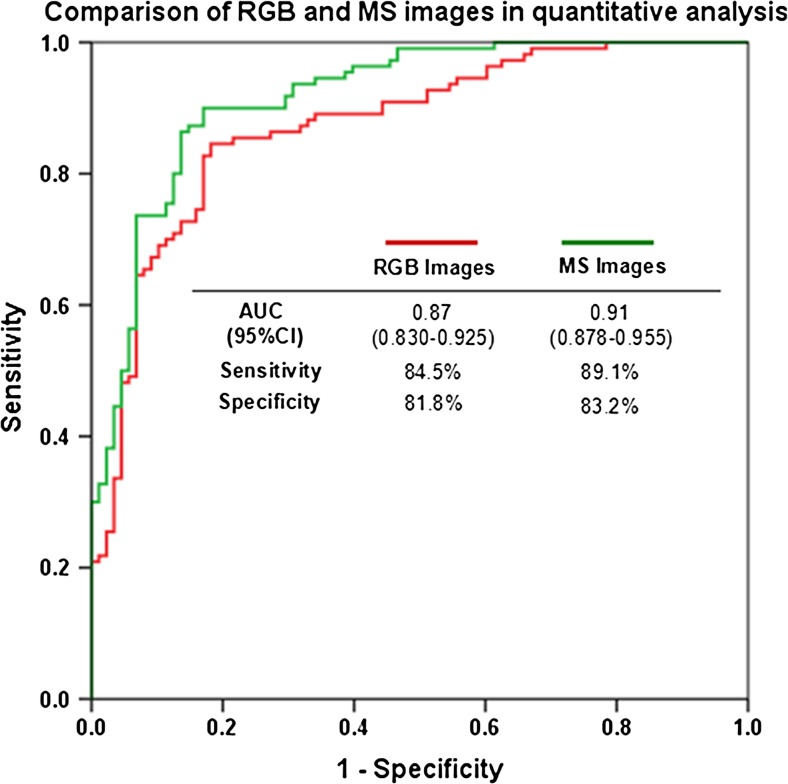
ROC analysis of quantitative outputs of RGB and MS images with *FISH* as gold standard. MS images have significantly increased area under the ROC curve, sensitivity, and specificity than the RGB images. The application of MS images in quantitative IHC could have better diagnostic performance in BC patients

Among the 86 specimens in this study, 45 cases had been previously identified by IHC as HER2-positive (IHC 2+ in 26 cases and IHC3+ in 19 cases), and specimens from these 45 cases were used for further validation studies by FISH and comparison studies among the three methods. FISH-positive results were found in 28 cases, including 11 of the 26 IHC 2+ cases and 17 of the 19 IHC 3+ cases. In order to compare the quantitative outputs of RGB and MS images, a ROC curve was generated comparing the diagnostic performance of RGB and MS images with FISH evaluation as gold standard. The ROC curve showed that the area under the curve was significantly greater in MS images (area under the curve (AUC) = 0.91; 95 % confidence interval (CI) 0.878–0.955; *P* < 0.001) than RGB images (AUC = 0.87; 95 % CI 0.830–0.925; *P* < 0.001). Moreover, the specificity and the sensitivity of RGB and MS images at optimal cutoffs were also illustrated in Fig. 4. The sensitivity was slightly higher in MS images (89.1 %) than in RGB images (84.5 %), and the specificity was also slightly higher in MS images (83.2 %) than in RGB images (81.8 %). The cutoffs of RGB and MS images were 74,074 and 86,420, respectively.

### Inter-observer consistency analysis in RGB and MS images

**Fig. 5:**
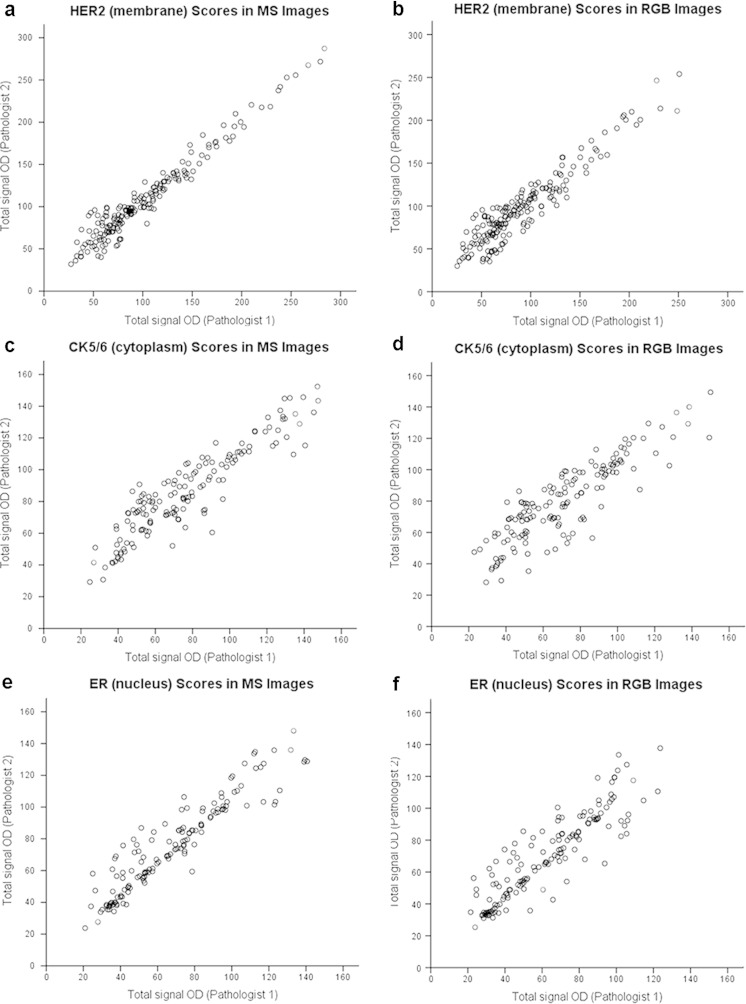
Inter-observer concordance between IHC quantitative analysis for MS and RGB images, shown as dotplots (Spearman’s rank correlation). **a**, **b** Correlations of membrane HER2 scores in MS versus RGB images (*r*_*s*_ = 0.94 vs. 0.89, all *P* < 0.001). **c**, **d** Correlations of cytoplasm CK5/6 scores in MS versus RGB images (*r*_*s*_ = 0.88 vs. 0.84, all *P* < 0.001). **e**, **f** Correlations of nucleus ER scores in MS versus RGB images (*r*_*s*_ = 0.94 vs. 0.89, all *P* < 0.001)

The agreement analysis on the studied biomarkers showed that the inter-observer consistency was better in MS images for membrane (HER2) (ICC = 0.95, *r*_s_ = 0.94, *P* < 0.001), cytoplasm (CK5/6) (ICC = 0.90, *r*_s_ = 0.88, *P* < 0.001), and nucleus (ER) (ICC = 0.94, *r*_s_ = 0.94, *P* < 0.001) than in RGB images for membrane (HER2) (ICC = 0.91, *r*_s_ = 0.89, *P* < 0.001), cytoplasm (CK5/6) (ICC = 0.85, *r*_s_ = 0.84, *P* < 0.001), and nucleus (ER) (ICC = 0.90, *r*_s_ = 0.89, *P* < 0.001) (Fig. 5).

#### Quantitative results of different images in relation to clinico-pathological characteristics

Forty-five HER2-positive expression cases (IHC 2+ in 26 cases and IHC3+ in 19 cases) were used to analyze the relationship of quantitative results of different images and clinico-pathological characteristics. As shown in Table 3, we did not find a statistically significant correlation between the total signal OD values in RGB images of HER2 and age, menopausal status, tumor size, lymph node status, histological grades, chemotherapy, and recurrence (*P* > 0.05). It was interesting to note that, by quantifying MS images, quantitative results of HER2 were correlated with lymph node status and histological grades; the total signal OD values were higher in lymph-node-positive than in lymph-node-negative tumors (*P* = 0.02) and in high-grade than in middle-grade tumors (*P* = 0.04).

**Table 3:** HER2 immunohistochemical score for RGB and MS images in relation to clinico-pathological characteristics

Variables	Total	HER-positive (*n*)	RGB images			MS images		
			No.	Total signal OD (×10^3^)	*P* value	No.	Total signal OD (×10^3^)	*P* value
Age (years)					0.36			0.34
<50	32	21	126	84.586 ± 40.154		126	96.149 ± 84.650	
>50	54	24	144	91.376 ± 75.847		144	100.192 ± 68.947	
Menopausal status					0.68			0.47
Premenopausal	29	22	132	87.019 ± 39.937		132	91.442 ± 43.603	
Postmenopausal	57	23	138	90.150 ± 76.720		138	96.634 ± 69.388	
Tumor size (cm)					0.17			0.14
T ≤ 5	70	30	180	89.421 ± 42.474		180	102.881 ± 44.085	
T > 5	16	15	90	97.146 ± 48.849		90	111.183 ± 51.048	
Lymph node status					0.06			**0.02**
Positive	36	25	150	96.097 ± 48.255		150	109.118 ± 73.246	
Negative	50	20	120	86.078 ± 35.243		120	90.013 ± 38.430	
Histological grades					0.46			**0.04**
І	17	6	36	59.678 ± 13.464		36	59.631 ± 18.893	
П	46	21	126	85.370 ± 50.951		126	92.379 ± 41.402	
Ш	23	18	108	91.265 ± 39.603^*^		108	102.995 ± 41.097^*^	
Chemotherapy					0.48			0.31
No	37	19	114	90.186 ± 19.246		114	92.851 ± 21.654	
Yes	49	26	156	85.279 ± 69.983		156	89.544 ± 29.129	
Recurrence					0.73			0.59
No	51	22	132	97.925 ± 31.951		168	104.835 ± 35.558	
Yes	35	23	138	99.435 ± 40.661		102	102.305 ± 42.811	

Data are presented as means ± standard deviation

*OD* optical density

* *P* < 0.05 compared with histological grade П

## Discussion

Quantitative pathology is the development trend in clinical diagnostic pathology, which requires two types of key technology to achieve this goal. First, the imaging technology for digital pathology with high definition, high resolution, and high contrast should be developed. Second, the acquisition and analysis technology with high precision, high throughput, and high sensitivity should also be developed. Focusing on common parameters in BC cell membrane, cytoplasm, and nucleus as the research object, this study attempted to validate a kind of novel and standard IHC digital imaging technique.

For imaging technology, compared with colorized digital camera or monochromatic camera, MS imaging can obtain the information on color spectrum distribution at each pixel of a color image, whereas conventional RGB imaging can only obtain information on specific color distribution of the three available channels (red, green, and blue) [13, 25], as demonstrated by spectral curves obtained in our results. Previous studies showed that MS imaging currently uses liquid crystal tunable filter (LCTF) technology to allow relatively narrow wavelength bands (10∼20 nm) to transmit wavelengths of light in the visible and near-infrared ranges, and spectral data of different wavelengths was acquired. It establishes a three-dimensional “cube,” with each spectrum corresponds to each pixel point in the cube, and classifies and separates the spectrum efficiently and accurately, which can significantly ameliorate the imaging process and suit multiple target imaging of both bright-field and fluorescence images. After the spectral data is acquired, a high-quality color image is generated from the image stack (or cube) and displayed [9, 10, 13]. The corresponding RGB and MS images of HER-2, CK5/6, and ER IHC staining in this work were acquired using RGB imaging and MSI system. The imaging results showed that the overall quality of MS images is improved significantly, with richer color information, higher resolution, and stronger contrast, which will help quantitative image analysis. As suggested by Boucheron et al. [26], MS images are better than the three standard bands of RGB images not only in color intensity, image resolution, and color contrast but also in additional useful information and image segmentation effect.

Previous applications of MSI in pathology mainly focused on analysis in routine hematoxylin-and-eosin images [26–28], and the application of this technology in IHC analysis has been on steady increase. Traditionally, IHC interpretation may be standardized using current digital imaging technology, avoiding the use of three- or four-point subjective scoring methods. Several studies have shown that bright-field RGB scores were difficult to count exactly, as the tumor cells always overlap one another [5, 6, 11, 29]. However, MSI can allow one to separate overlapping chromogens and unmix multiple chromogens in multicolor IHC [11, 25, 30].

Accurate signal unmixing are prerequisites for valid quantitation of IHC-positive signal intensities. Quantitation typically requires some spatial manipulations such as segmentation of morphologic tissue and identification of subcellular compartments [30]. In order to effectively analyze captured images, the software employed must be able to extract chromogenic intensity information from the images [5, 30]. In our study, we observed the unmixed effect of different chromogens in RGB and MS images by CRi Nuance software package. It is simple to obtain clearer separated quantitative images and better segmentation accuracy from unmixing MS images of each marker than RGB images, no matter whether positive choromogens occurred in the membrane, the cytoplasm, or the nucleus. In order to visualize the co-localization better, these unmixed images can be recolored and layered together as composite images, rendering a simulated bright-field view. Therefore, the richer color information contained in MS images is more suitable for image quantitative analysis than conventional RGB images.

In BC patients, accurate assessment of hormone receptor (HR), HER2 status, and CK5/6 of the tumor is critical for defining the molecular subtypes and predicting response to systemic therapies [21, 31, 32]. We measured the total signal OD values in RGB and MS images of membrane (HER2), cytoplasm (CK5/6), and nucleus (ER) in invasive BC using CRi Nuance software package. The results showed that the total signal OD values were higher in MS images than the corresponding RGB images, and the differences achieved statistical significance (*P* < 0.01, for all). This possibly due to MS images contain stronger color intensities and more precise segmented capacities. However, whether high numerical quantitative results can obtain high diagnostic coincidence rate remains to be seen. Our previous studies also showed that MSI could be applied to QD-based fluorescence imaging to help improve tumor classifications and predict tumor prognosis [15].

In the validation study using HER2 as a model, we compared the MS images analysis outputs, RGB images analysis outputs, and FISH results. ROC curve analysis, a useful method to evaluate the performance of diagnostic systems, showed greater AUC, higher specificity, and sensitivity in MS images than RGB images. These results indicate that the MS images could facilitate better diagnostic performance than RGB images. Huang et al. [13] demonstrated that MSI technology is reliable for objective and high-throughput biomarker quantitation and colocalization study using chromogenic multiplexed immunohistochemistry.

By measuring RGB images, we found no significant difference between HER2 expression and age, menopausal status, tumor size, lymph node status, histological grades, chemotherapy, recurrence (*P* > 0.05). However, by quantifying MS images, we first reported that quantitative results of HER2 expression had relation to the lymph node status and histological grades of invasive BC; the total signal OD values were higher in lymph-node-positive than in lymph-node-negative tumors and in high-grade tumors than in middle-grade tumors (*P* = 0.02 and 0.04). It seems that more information could be provided by quantifying MS images of the protein expression in relation to differentiation of invasive BC.

In addition, consistency analysis results showed that quantitative evaluation of MS images of membrane (HER2), cytoplasm (CK5/6), and nucleus (ER) reached a better inter-observer agreement and reproducibility than RGB images. Although some factors may influence inter-observer variability such as application of software and observers themselves, this indicates that quantitative estimation of MS images of specific biomarker may be sufficient for reliable and robust measurement of IHC in invasive breast carcinoma.

Our study has several limitations. First, although 86 BC specimens were enrolled in our study, it finally included only 45 HER2-positive, 35 CK5/6-positive, and 40 ER-positive cases, which could result in possible sample selection bias and would have been more justifiable with more subjects. Second, the objective evaluation including mean squared error (MSE), peak signal-to-noise ratio (PSNR), and structural similarity (SSIM) [33] of different image types was not performed in our results on the ground that we primarily focused on comparing their sensitivity, specificity, and application effect in automated quantitative analysis of IHC. Additional studies are needed to identify the advantages of spectral image by calculating accurately the objective evaluation index of the image. Third, the analysis software used in this study could not eliminate the interference caused by three biomarkers expressed in the stroma, although such expression in the stroma is very little, and we are comparing two kinds of digital images under the same conditions. Fourth, despite higher sensitivity and specificity and more accurate quantification in MS images than conventional RGB images, we did not assess the relationship between quantitative analysis of different images and prognosis of invasive BC. Further prospective clinical studies are required to validate the advantages of MS images.

In conclusion, our study demonstrates the potential of MSI in quantitative analysis of IHC and highlights the inherent advantages of more accurate spectra quantification of MS images over conventional RGB images. Based on these advantages, an improved technical platform for IHC digital imaging could be developed for computer-aided quantitative digital pathology.
